# HIV-1 and HIV-2 exhibit similar mutation frequencies and spectra in the absence of G-to-A hypermutation

**DOI:** 10.1186/s12977-015-0180-6

**Published:** 2015-07-10

**Authors:** Jonathan M O Rawson, Sean R Landman, Cavan S Reilly, Louis M Mansky

**Affiliations:** Institute for Molecular Virology, University of Minnesota, Minneapolis, MN USA; Department of Diagnostic and Biological Sciences, School of Dentistry, University of Minnesota, Minneapolis, MN USA; Department of Microbiology, University of Minnesota, Minneapolis, MN USA; Molecular, Cellular, Developmental Biology and Genetics Graduate Program, University of Minnesota, Minneapolis, MN USA; Department of Computer Science and Engineering, University of Minnesota, Minneapolis, MN USA; Division of Biostatistics, School of Public Health, University of Minnesota, Minneapolis, MN USA

**Keywords:** HIV-1, HIV-2, Viral mutagenesis, Viral evolution, Hypermutation

## Abstract

**Background:**

Human immunodeficiency virus type 2 (HIV-2) is often distinguished clinically by lower viral loads, reduced transmissibility, and longer asymptomatic periods than for human immunodeficiency virus type 1 (HIV-1). Differences in the mutation frequencies of HIV-1 and HIV-2 have been hypothesized to contribute to the attenuated progression of HIV-2 observed clinically.

**Results:**

To address this hypothesis, we performed Illumina sequencing of multiple amplicons prepared from cells infected with HIV-1 or HIV-2, resulting in ~4.7 million read pairs and the identification of ~200,000 mutations after data processing. We observed that: (1) HIV-2 displayed significantly lower total mutation, substitution, and transition mutation frequencies than that of HIV-1, along with a mutation spectrum markedly less biased toward G-to-A transitions, (2) G-to-A hypermutation consistent with the activity of APOBEC3 proteins was observed for both HIV-1 and HIV-2 despite the presence of Vif, (3) G-to-A hypermutation was significantly higher for HIV-1 than for HIV-2, and (4) HIV-1 and HIV-2 total mutation frequencies were not significantly different in the absence of G-to-A hypermutants.

**Conclusions:**

Taken together, these data demonstrate that HIV-2 exhibits a distinct mutational spectrum and a lower mutation frequency relative to HIV-1. However, the observed differences were primarily due to reduced levels of G-to-A hypermutation for HIV-2. These findings suggest that HIV-2 may be less susceptible than HIV-1 to APOBEC3-mediated hypermutation, but that the fidelities of other mutational sources (such as reverse transcriptase) are relatively similar for HIV-1 and HIV-2. Overall, these data imply that differences in replication fidelity are likely not a major contributing factor to the unique clinical features of HIV-2 infection.

**Electronic supplementary material:**

The online version of this article (doi:10.1186/s12977-015-0180-6) contains supplementary material, which is available to authorized users.

## Background

Human immunodeficiency virus type 1 (HIV-1) infects approximately 35 million individuals worldwide and has resulted in about 39 million deaths since the onset of the AIDS pandemic (http://www.unaids.org). Within infected individuals, HIV-1 undergoes rapid genetic diversification, promoting the acquisition of drug resistance, evasion of the host immune response, and alterations in cell tropism. Genetic diversification is, in turn, driven by large population sizes and high rates of replication, recombination, and mutation. HIV-1 has been found to mutate on the order of 10^−4^ to 10^−5^ mutations/base pair/cycle (m/bp/c), corresponding to ~0.1–1 mutations per genome synthesized [1–5]. HIV-1 thus mutates about 10,000–100,000 times faster than eukaryotic genomic DNA [6]. Multiple viral and cellular factors influence both the rates and types of mutations produced during viral replication. Reverse transcriptase (RT) is likely a major generator of mutations in vivo, as it is tremendously error-prone in vitro (mutation rates >10^−4^ m/bp/c), primarily due to a lack of proofreading activity [7, 8]. RNA polymerase II can also introduce mutations when transcribing the viral RNA genome, but less than that of RT [3]. Cellular DNA polymerases can introduce mutations when replicating the proviral DNA integrated into the cellular genome; however, these enzymes have been assumed to minimally contribute to HIV-1 variation due to the high fidelity of genomic DNA replication [6]. In addition, apolipoprotein B mRNA-editing enzyme catalytic polypeptide-like 3 (APOBEC3) proteins can induce G-to-A hypermutation, particularly in the absence of Vif [9, 10]. APOBEC3-mediated hypermutation is often lethal to virus replication, but accumulating evidence suggests that APOBEC3 proteins can in some cases promote genetic diversification and the evolution of new variants conferring drug resistance or altered cell tropism [11–15]. Other factors, such as dNTP pool levels [16], other viral proteins (Vpr) [17, 18], and cell type [19] have been shown to influence HIV-1 mutagenesis as well.

Relative to HIV-1, human immunodeficiency virus type 2 (HIV-2) infection is often marked clinically by lower viral loads, reduced transmissibility, and longer asymptomatic periods in infected individuals [20, 21]. Differences in the mutation frequencies of HIV-1 and HIV-2 have been hypothesized to contribute to the attenuated progression of HIV-2 observed clinically. Specifically, a lower mutation rate for HIV-2 would be expected to limit genetic diversification, which could in turn reduce viral fitness and/or attenuate viral pathogenicity. Consistent with this hypothesis, viral variants with increased replication fidelity have been shown to result in impaired viral fitness and virulence in many other RNA viruses [22–26]. In further support of this hypothesis, HIV-2 has been found to evolve less quickly than HIV-1 [27, 28], though one report has found the opposite [29]. However, it should be noted that evolutionary rates depend on a wide variety of factors besides the mutation rate, such as replication rate, population size, and selective pressures. In addition, the HIV-2 RT contains a highly conserved V75I polymorphism, a drug resistance-associated mutation in HIV-1 that improves RT fidelity [30, 31]. Lastly, HIV-2 has been shown to be less sensitive than HIV-1 to APOBEC3G activity, potentially diminishing the contribution of APOBEC3G to viral mutagenesis [32].

In order to compare mutation frequencies and spectra between HIV-1 and 2, we performed Illumina high-throughput sequencing of proviral DNA from cells infected with HIV-1 or 2. We found that HIV-2 displayed lower total, substitution, and transition mutation frequencies than HIV-1, particularly due to reduced levels of G-to-A transition mutations. We also observed low-level G-to-A hypermutation in both HIV-1 and HIV-2 that was consistent with the activity of APOBEC3 proteins. Intriguingly, HIV-2 demonstrated significantly lower levels of G-to-A hypermutation than HIV-1. After exclusion of G-to-A hypermutants, total mutation frequencies were not significantly different between HIV-1 and HIV-2. Together, these data support the conclusion that differences in general replication fidelity are likely not a primary driver of the unique clinical features of HIV-2 infection.

## Results

### Characterization of background errors induced by PCR and Illumina sequencing

**Figure 1:**
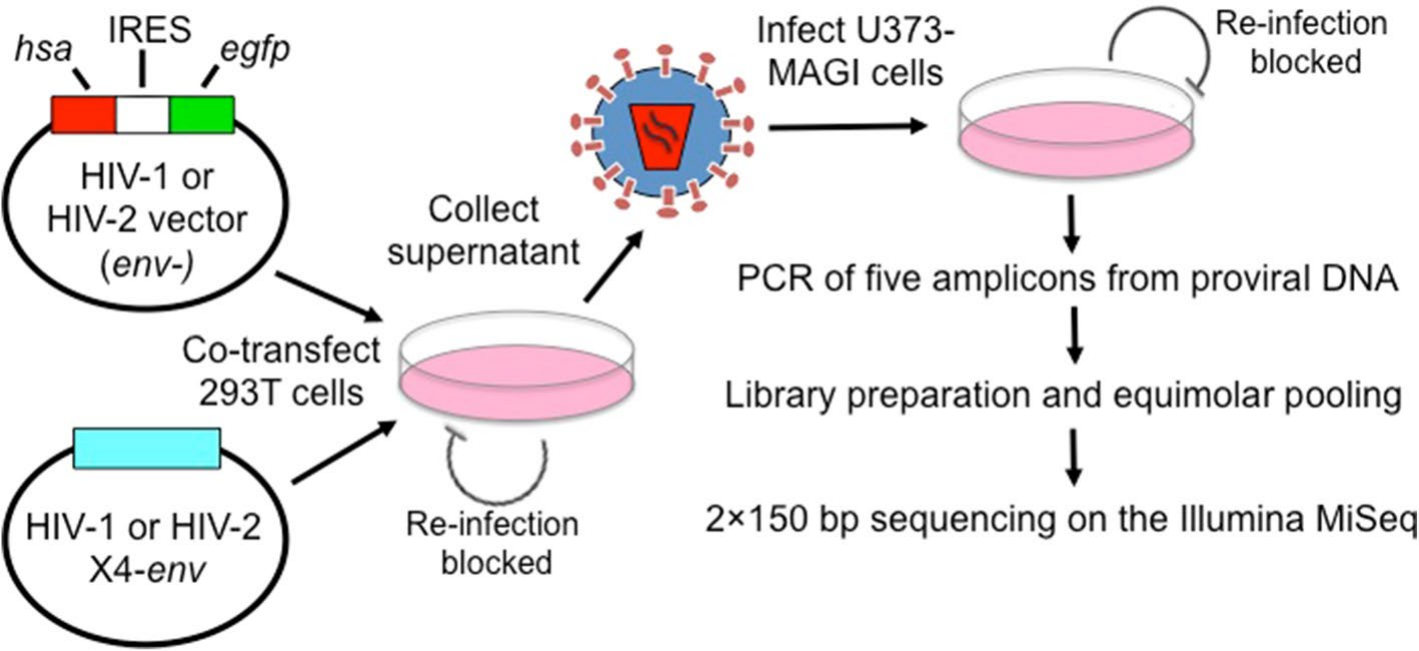
Experimental strategy for investigating HIV-1 and HIV-2 mutagenesis by Illumina DNA sequencing. Vector virus stocks were produced by co-transfecting 293T cells with HIV-1 or HIV-2 Env-deficient vectors and HIV-1 or HIV-2 CXCR4-tropic Env expression constructs. Virus stocks were concentrated, DNase I-treated to reduce plasmid carryover, and titered in U373-MAGI cells. To prepare samples for Illumina sequencing, 1 × 10^6^ U373-MAGI cells were infected at an MOI of 1.0, generating approximately 1 × 10^6^ proviruses per experimental replicate. This assay represents a single round of viral replication, as producer cells and target cells cannot be re-infected, due to a lack of receptor or Env expression, respectively. Polymerase chain reaction (PCR) of five amplicons (Gag, Vif, HSA, EGFP-1, and EGFP-2) was performed from the proviral DNA. Amplicons from the HIV-1 and HIV-2 proviral DNAs were either identical (HSA, EGFP-1 and 2) or homologous (Gag and Vif) in sequence. The EGFP-1 and EGFP-2 amplicons represent non-overlapping segments of the *egfp* gene. Sequencing libraries were prepared from the amplicons, pooled in an equimolar fashion to normalize coverage, and subjected to 2 ×150 bp sequencing on the Illumina MiSeq, generating approximately 4.7 million read pairs after data processing.

In order to compare mutation frequencies and spectra between HIV-1 and HIV-2, single cycle infections with HIV-1 or HIV-2 were performed at a high MOI (1 million U373-MAGI-X4 cells per replicate infected at an MOI of 1.0, see “Methods”; Figure 1). In this assay, producer cells cannot be re-infected due to a lack of the appropriate receptor and co-receptor, and target cells likewise cannot be re-infected due to disruption of the *env* genes in the HIV-1 and HIV-2 vectors. Genomic DNA was purified from infected cells and first subjected to quantitative PCR (qPCR) in order to determine the level of plasmid carryover from transfections. Plasmid carryover was quantified either by: (1) determining the plasmid backbone copy number (by measuring the ampicillin resistance gene) and dividing by the proviral copy number, or (2) determining the proviral copy number from heat-inactivated viral infections and dividing by the proviral copy number from un-treated infections (see “Methods”). We found that the level of plasmid carryover for HIV-1 was 0.2% when measured by either method, while the level of carryover was 2.8 or 1.4% for HIV-2, depending on the approach used (Additional file 1: Table S1). The significantly higher level of plasmid carryover for HIV-2 likely reflects the reduced infectivity of HIV-2 viral stocks, which resulted in larger volumes of viral stocks being used during infection. These results are comparable to those obtained in another study [5] and are too low to significantly impact measured mutation frequencies. Next, amplicons were prepared from proviral DNA for Illumina sequencing. In total, 12 samples were analyzed—three experimental replicates each of HIV-1, HIV-2, and HIV-1 and HIV-2 plasmid amplifications as controls to determine levels of background errors. Further, for each sample, five amplicons were prepared (Gag, Vif, HSA, EGFP-1, and EGFP-2), representing a mixture of viral (Gag, Vif) and marker (HSA, EGFP-1, EGFP-2) gene targets. Libraries were prepared individually from samples in order to prevent inter-sample recombination during library construction. Following this, all libraries were pooled and subjected to 2 × 150 paired-end sequencing on the Illumina MiSeq, resulting in ~4.7 million total read pairs after processing, or an average of ~79,000 read pairs/amplicon/sample (Additional file 2: Table S2). After stringent filtering of Illumina data, the mutation frequencies (expressed as mutations per base pair, or m/bp) were determined for all samples, both in terms of total mutations and every possible subdivision (i.e. substitutions, transitions, transversions, etc.). Mutation counts, frequencies, and relative percentages are listed in Additional file 3: Dataset S1, both combined across all five amplicons and separated by amplicon.

**Figure 2:**
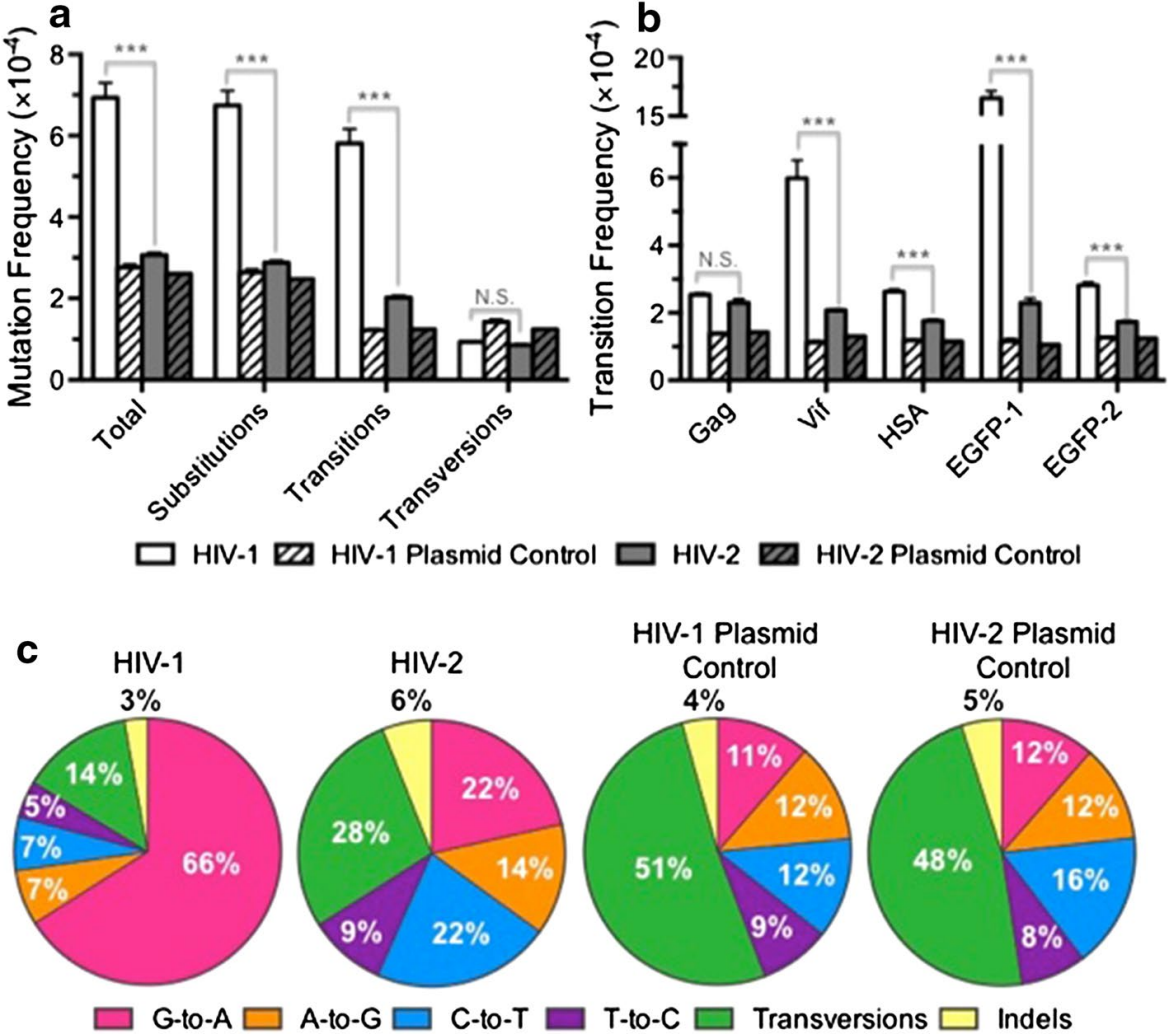
HIV-2 has a lower mutation frequency and distinct mutation spectrum relative to HIV-1. **a** Mutation frequency analysis. Mutation frequencies were calculated by dividing the number of mutations by the number of reference bases (mutations + wild-type bases) and are expressed as mutations/bp, or m/bp. Mutation frequencies were determined for HIV-1 and HIV-2, as well as for plasmid controls to assess background error levels. **b** Transition frequency analysis. Transition frequencies were compared across the five different amplicons subjected to Illumina DNA sequencing. **c** Mutation spectra analysis. Mutation spectra were determined by dividing the frequency of each type of mutation by the total mutation frequency, with the results expressed as a percentage of total mutations. Data in *all panels* represent the mean of three experimental replicates, with *error bars* indicating the standard deviation. *Asterisks* denote statistically significant differences between HIV-1 and 2 (*p < 0.05, ***p < 0.001, *N.S.* not significant). The actual numbers of read pairs, mutations, and reference bases are listed in Additional file 3: Dataset S1.

After sequencing, the first objective was to utilize the plasmid controls to characterize the frequencies and spectra of background errors (i.e. errors from PCR or sequencing) in order to determine the extent to which biological mutations could be detected above the level of the background. The average mutation frequencies of the plasmid controls were 2.8 (HIV-1) and 2.6 (HIV-2) ×10^−4^ m/bp (Figure 2a; Additional file 3: Dataset S1), consistent with a recent investigation into background error during amplicon sequencing on the Illumina MiSeq [33]. Most of the background errors observed were substitutions, with insertions and deletions comprising only 4.3–4.9% of total mutations. Of the substitutions, transversions and transitions were observed at similar frequencies: 1.2–1.4 × 10^−4^ m/bp for transversions and ~1.2 × 10^−4^ m/bp for transitions, such that each category composed ~half of all mutations (Figure 2c). However, as described in “Methods”, plasmid error hotspots (i.e. positions highly prone to background error) were masked prior to this analysis, and in the absence of such masking transversions occurred ~2.5-fold more often than transitions. Thus, our findings are consistent with previous reports that have observed a bias of Illumina sequencing (using a variety of different platforms and library preparation methods) toward transversion types [33–36]. Interestingly, among the eight possible transversion types, background transversion errors were non-randomly distributed (Additional file 4: Figure S1). Specifically, background transversions were strongly biased toward C-to-A and G-to-T transversions, which composed 74% (HIV-1) or 73% (HIV-2) of all transversions. C-to-A and G-to-T are reciprocal mutational types, and these mutations may be due to oxidative conversion of guanine to 8-oxoguanine or due to Illumina imaging artifacts (see “Discussion”). Background errors were distributed quite evenly across the five amplicons (Figure 2b), demonstrating that no individual amplicon was particularly prone to background errors.

In addition to analyzing background error frequencies and spectra, the frequency of intra-sample recombination due to PCR was also examined. While PCR-mediated recombination is not known to be mutagenic, recombination could affect associations between mutations. We attempted to minimize recombination from PCR by stopping reactions after 30 cycles of amplification, corresponding to the ~end of the log-linear phase of amplification, after which recombination occurs at a much higher rate due to saturation of dNTPs and primers [37–39]. Intra-sample recombination frequencies were determined using genetic markers that were incorporated into the plasmid control amplifications (see “Methods”). Under these amplification conditions, intra-sample recombination was observed at frequencies of ~2–3% of the maximum observable in the assay, demonstrating that recombination from PCR was relatively rare (Additional file 5: Table S3).

We next compared the mutation frequencies and spectra of the HIV-1 and 2 biological samples to that of the plasmid controls in order to determine which types of mutations could be detected above background levels. HIV-1 and HIV-2 exhibited average mutation frequencies of 6.9 (HIV-1) and 3.1 (HIV-2) × 10^−4^ m/bp (Figure 2a; Additional file 3: Dataset S1). The total mutation frequency of HIV-1 was significantly higher than the corresponding plasmid control (*p* < 0.001). In contrast, the HIV-2 total mutation frequency was not significantly higher than the plasmid control (*p* = 0.40). Upon separating out the major classes of mutations, we found that this was primarily due to high levels of transversions in the plasmid controls. Transversions occurred at frequencies of 1.4 (HIV-1) or 1.2 (HIV-2) × 10^−4^ m/bp in the plasmid controls (Figure 2a), values that were not significantly different from that of the biological samples. Similar to transversions, insertion frequencies did not significantly vary between biological samples and plasmid controls, and deletion frequencies were only significantly higher than plasmid for HIV-2 (*p* = 0.02, Additional file 3: Dataset S1). However, insertions and deletions had little impact on overall mutation frequencies because they were much less frequent (3–6% of total mutations) than substitutions (Figure 2c). Notably, although the HIV-2 total mutation frequency was not significantly higher than its corresponding plasmid control, transition frequencies [5.8 (HIV-1) and 2.0 (HIV-2) × 10^−4^ m/bp] were significantly higher than the plasmid controls for both viruses (HIV-1: *p* < 0.001; HIV-2: *p* = 0.038). These differences were also reflected in the mutation spectra, as transitions comprised 84% (HIV-1) or 66% (HIV-2) of total mutations in the biological samples but only 44% (HIV-1) or 48% (HIV-2) in the plasmid controls (Figure 2c). Importantly, many previous reports have demonstrated that transitions predominate during the replication of HIV-1, generally comprising 70–90% of all substitutions [1–4, 17, 19, 40]. Thus, considering that transitions are more relevant to HIV-1 replication and that they were detected at levels significantly higher than the background, most downstream analyses focused on transition mutational types.

### HIV-2 exhibits a lower mutation frequency and an altered mutation spectrum relative to that of HIV-1

We next compared HIV-1 and HIV-2 mutation frequencies in order to test our initial hypothesis that HIV-2 would display a lower mutation frequency than HIV-1. We found that HIV-1 had a significantly higher total mutation frequency, as well as higher frequencies of substitutions and transitions, than HIV-2 (relative differences of 2.3, 2.3, and 2.9-fold, respectively; all *p* values <0.001) (Additional file 3: Dataset S1; Figure 2a). In contrast, the levels of transversion mutations were not significantly different between HIV-1 and HIV-2 (*p* = 0.32). The observed differences in transition frequencies were primarily due to an approximately 6.9-fold higher frequency (*p* < 0.001) of G-to-A transition mutations with HIV-1 than with HIV-2 (Additional file 3: Dataset S1). Less striking, but statistically significant, differences were observed for several other types of transitions as well. HIV-1 displayed a 1.1-fold higher frequency of A-to-G transitions (*p* = 0.049) than HIV-2, while HIV-2 displayed a 1.5-fold higher frequency of C-to-T transitions than HIV-1 (*p* = 0.0019). The frequency of T-to-C transitions was not significantly different between HIV-1 and HIV-2. Consistent with these results, the mutation spectrum of HIV-1 was much more heavily biased toward G-to-A transition mutations than for HIV-2 (66 vs. 22% of total mutations, Figure 2c). Overall, these data demonstrate that the HIV-1 mutation frequency is significantly higher than that of HIV-2, predominantly due to substantially higher levels of G-to-A transition mutations.

### Mutation frequencies vary across amplicons for HIV-1 and HIV-2

We hypothesized that specific amplicons might be more or less error-prone than others due to features of the primary sequence (such as homopolymeric runs), secondary structures, or the relative positioning of the amplicon within the viral genome. To address this, transition frequencies were compared across the five amplicons analyzed in this study. As previously indicated, transitions in the plasmid controls were distributed relatively evenly across the five amplicons, demonstrating that no individual amplicon was particularly prone to background transition mutations (Figure 2b). In contrast, for HIV-1, it was found that most transitions were concentrated in the EGFP-1 amplicon and, to a lesser extent, the Vif amplicon (Figure 2b). The EGFP-1 and Vif amplicons together accounted for about 74% of all transitions in HIV-1. The relative order of transition frequencies among the amplicons was found to be EGFP-1 > Vif > EGFP-2 ≈ HSA ≈ Gag, with all indicated differences between amplicon pairs being statistically significant (*p* < 0.001). Since G-to-A transitions predominated for HIV-1, the observed differences could potentially be explained by varying nucleoside content between amplicons. However, the amplicons contained relatively similar frequencies of deoxyguanosine, ranging from 19.6 to 31.2%. Further, the Vif amplicon actually contained the lowest deoxyguanosine content of the five amplicons, despite having the second highest transition frequency. For HIV-2, transition frequencies were much more evenly distributed between amplicons than for HIV-1, with a maximal difference of ~1.3-fold (between Gag and EGFP-2). We also compared transition frequencies between HIV-1 and HIV-2 at the level of individual amplicons. HIV-1 was found to have a higher frequency of transitions than HIV-2 in all amplicons except Gag (*p* = 0.12 for Gag; *p* < 0.001 for all other amplicons); however, the greatest differences were observed in the EGFP-1 and Vif amplicons (7.2 and 2.9-fold differences, respectively).

### Detection of G-to-A hypermutation in HIV-1 and HIV-2

Considering the high levels of G-to-A transition mutations (particularly for HIV-1) that we observed (Figure 2c), we next investigated whether HIV-1 or 2 displayed evidence of G-to-A hypermutation. G-to-A hypermutation could potentially arise from the activity of APOBEC3 proteins, though relatively little APOBEC3 activity was expected in this particular experimental system (see “Discussion”). In these analyses, hypermutants were defined as read pairs (~120 bp in length) that contained two or more mutations of the same type within the read pair. In addition to analyzing G-to-A hypermutants, we determined frequencies of other possible types of transition hypermutants (A-to-G, C-to-T, and T-to-C) as well, as HIV-1 A-to-G hypermutants have occasionally been reported in the literature [4, 41]. All hypermutant counts and frequencies (defined as hypermutant read pairs/all read pairs) are tabulated in Additional file 6: Dataset S2, either combined across all amplicons or separated by amplicon.

**Figure 3:**
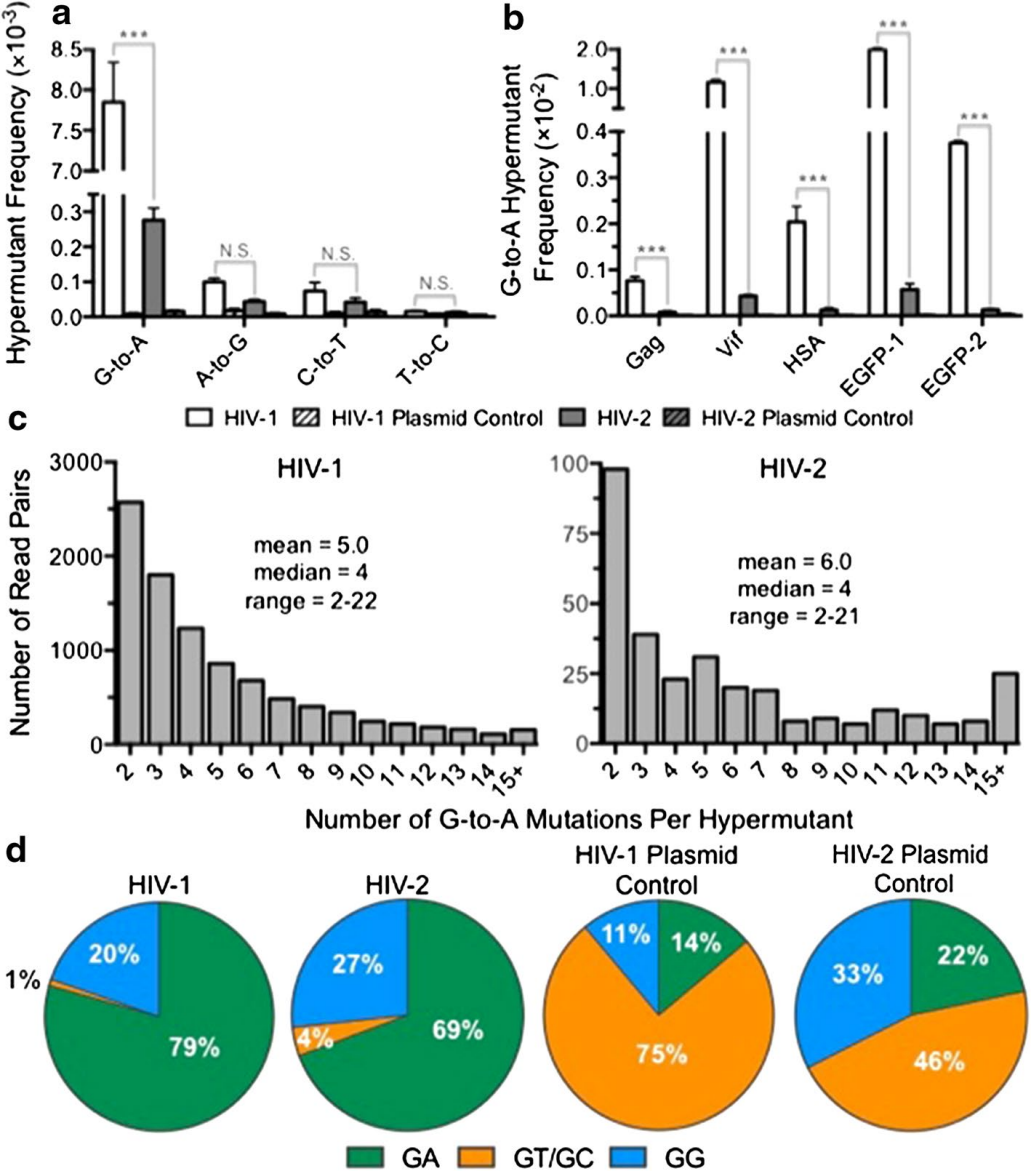
HIV-1 demonstrates higher G-to-A hypermutant frequencies than HIV-2. **a** The frequencies of each type of transition hypermutant were compared between HIV-1, HIV-2, and the plasmid controls. For this analysis, hypermutants were defined as read pairs containing two or more mutations of the indicated type within an individual read pair (approximately 120 bp in length). The frequency of hypermutation was then calculated by dividing the number of hypermutant read pairs by all read pairs. **b** The frequency of G-to-A hypermutation was compared across all five amplicons examined by Illumina DNA sequencing. **c** The degree of G-to-A hypermutation was analyzed by determining the numbers of G-to-A mutations within hypermutant read pairs. **d** The dinucleotide context of G-to-A mutations from G-to-A hypermutants was determined and expressed as a percentage of the total. Data in *panels* **a**, **b**, and **d** represent the mean of three experimental replicates, with *error bars* representing standard deviation, while data in *panel* **c** represent the total (i.e. compiled) data. *Asterisks* denote statistically significant differences between HIV-1 and 2 (*p < 0.05, ***p < 0.001, *N.S.* not significant). The actual numbers of G-to-A hypermutant read pairs and reference read pairs are listed in Additional file 6: Dataset S2.

In HIV-1, the frequency of G-to-A hypermutants was approximately 7.9 × 10^−3^ (or ~1 of 127 read pairs), while in HIV-2 the frequency was much lower, approximately 2.8 × 10^−4^ (or 1 of 3,623 read pairs) (Figure 3a). The average G-to-A hypermutation frequencies were about 1,060-fold higher for HIV-1 or about 17-fold higher for HIV-2 as compared to their corresponding plasmid controls, respectively. HIV-1 and HIV-2 G-to-A hypermutant frequencies were significantly higher than the plasmid controls in all five amplicons examined (*p*-values ranging from <0.001 to 0.01). The G-to-A hypermutation frequency was much higher (i.e., about 28-fold) in HIV-1 than observed in HIV-2 (*p* < 0.001), demonstrating that HIV-2 was less susceptible to G-to-A hypermutation in this experimental system. This trend was significant in all five amplicons examined (*p* < 0.001). Other types of transition hypermutants were also observed (Figure 3a), but they were far less frequent than G-to-A hypermutants and were not observed at levels significantly higher than that of the plasmid controls. In HIV-1, G-to-A hypermutants comprised about 98% of all transition hypermutants, indicating a clear dominance over other possible types of transition hypermutants. When subdivided by amplicon, the distribution of HIV-1 G-to-A hypermutation was found to resemble that of all transitions (Figure 2b), with most G-to-A hypermutation concentrated in the EGFP-1 and Vif amplicons (Figure 3b). In fact, after removal of G-to-A hypermutants, transition frequencies were very consistent across all five amplicons, demonstrating that G-to-A hypermutants were responsible for the elevated transition frequencies in EGFP-1 and Vif (Additional file 7: Figure S2). The overall observed trend of G-to-A hypermutation among amplicons in HIV-1 was EGFP-1 > Vif > EGFP-2 > HSA > Gag (all *p* values < 0.001). Despite being much less frequent, HIV-2 G-to-A hypermutants were also concentrated mainly in the EGFP-1 and Vif amplicons, although differences between amplicons did not reach statistical significance. These observations indicate that G-to-A hypermutation can significantly vary between different genes and even between different regions of the same gene (i.e. EGFP-1 vs. EGFP-2).

G-to-A hypermutation was further analyzed by determining the number of G-to-A mutations per hypermutant read pair. Most G-to-A hypermutants contained low numbers of G-to-A mutations (medians of 4 for both viruses), as expected for these short (~120 bp) read pairs (Figure 3c). However, a minority of G-to-A hypermutants contained high numbers of G-to-A mutations (maxima of 22 and 21 for HIV-1 and HIV-2, respectively). In contrast to the biological samples, the ultra-rare G-to-A hypermutants observed in the plasmid controls nearly all contained only two G-to-A mutations (one triple G-to-A mutant was observed for the HIV-2 plasmid). Next, the dinucleotide contexts of G-to-A mutations in hypermutants were analyzed, as APOBEC3 proteins are strongly associated with GG or GA dinucleotide contexts [42–45], depending on the specific APOBEC3 family member. Most G-to-A mutations were found within the GA dinucleotide context for both viruses, and, strikingly, only 1% (HIV-1) or 4% (HIV-2) were found to occur in either the GT or GC sequence contexts (Figure 3d). These dinucleotide contexts were markedly different from the contexts of the rare G-to-A hypermutants identified in the plasmid controls (Figure 3d) as well as from the contexts of single G-to-A mutants (Additional file 8: Figure S3). Although the dinucleotide context appears to differ between the HIV-1 and HIV-2 plasmid controls, it should be noted that these contexts are based on extremely low numbers of hypermutants for the plasmids (10 for HIV-1 and 17 for HIV-2). The observed bias in the virological samples was not caused by a higher prevalence of the GA dinucleotide (relative to GG, GC, and GT) within the amplicon sequences, as GA dinucleotides only accounted for 24% of all guanine-containing dinucleotides. Taken together, these findings support the conclusion that the low level of G-to-A hypermutation observed was primarily caused by one or more APOBEC3 family members, despite the presence of Vif in our experimental system.

The patterns of G-to-A hypermutation were further characterized by investigating whether G-to-A mutations in hypermutants occurred at hotspots and, if so, whether sequence preferences (beyond the dinucleotide context) could be identified. In this analysis, we defined G-to-A hypermutation hotspots as statistical upper outliers using the 1.5× interquartile range (IQR) rule. Using this criterion, 18 (HIV-1) or 16 (HIV-2) hotspots for G-to-A hypermutation were identified, all of which were located in the Vif or EGFP-1 amplicons (Additional file 9: Table S4). Of these, 14 (13 in EGFP-1 and 1 in Vif) were shared between HIV-1 and HIV-2, suggesting a common mechanism of mutation. Because specific APOBEC3 proteins have been shown to exhibit additional sequence preferences at the −1 and +2 positions (relative to the mutated guanine), sequence logos for total G-to-A hypermutation hotspots, GA context hotspots, and GG context hotspots were generated. However, statistically significant preferences at other positions were not observed for HIV-1 or HIV-2 in any of these categories. Furthermore, in order to gauge the potential effects of hypermutation on protein expression and/or function, the coding changes introduced at G-to-A hypermutation hotspots were examined (Additional file 9: Table S4). G-to-A hypermutation did not result in the introduction of premature stop codons at any of the examined hotspots. Missense mutations were typically R-to-K (semi-conservative), D-to-N (semi-conservative), or E-to-K (non-conservative); however, other types were occasionally observed. Interestingly, several of the Vif mutations occurring in G-to-A hypermutants at these hotspots have been previously characterized (see “Discussion”) [15].

### HIV-1 and HIV-2 mutation frequencies and spectra are not significantly different in the absence of G-to-A hypermutants

**Figure 4:**
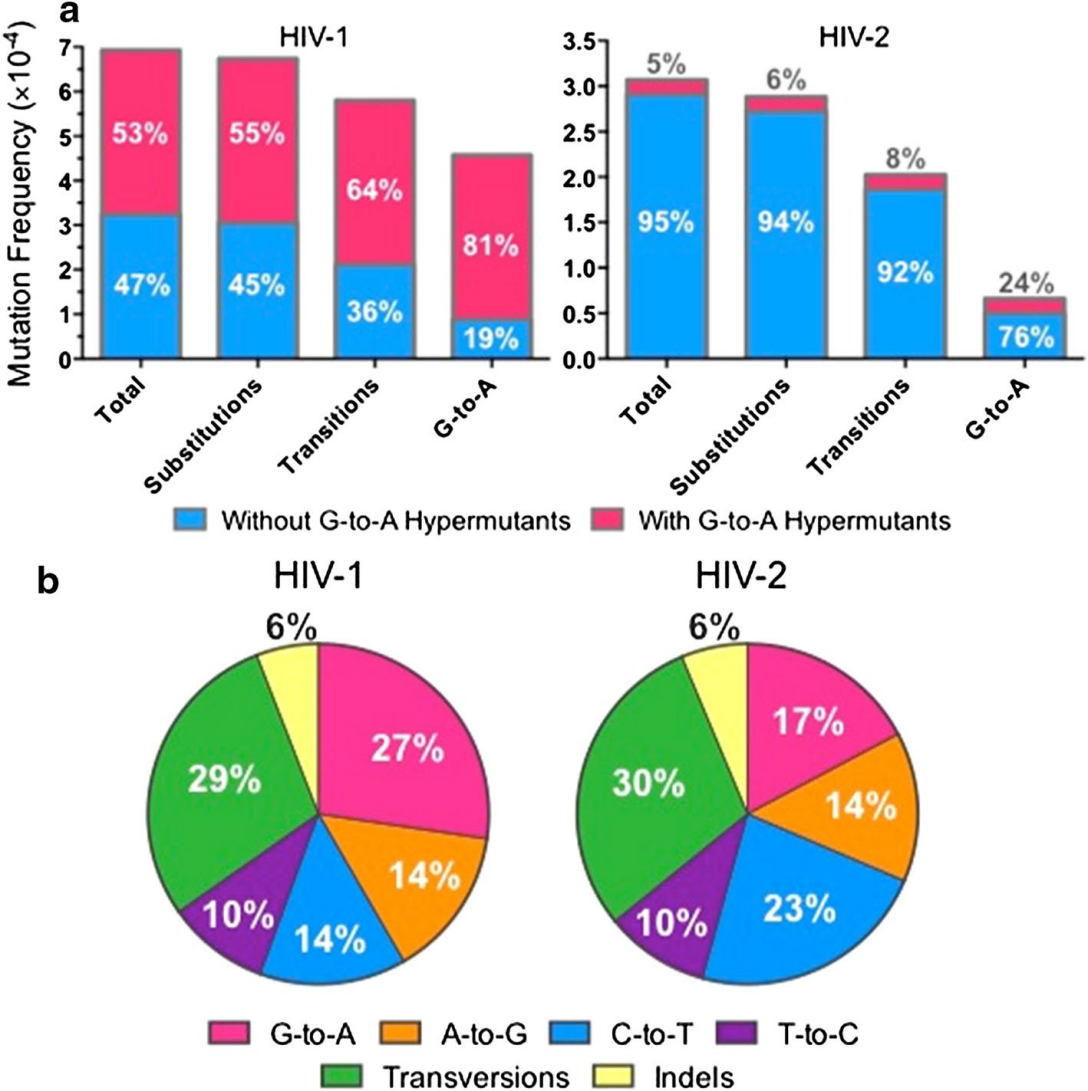
HIV-1 and HIV-2 mutation frequencies and spectra are similar in the absence of G-to-A hypermutation. **a** Analysis of mutation frequency in the absence of G-to-A hypermutation. HIV-1 and HIV-2 mutation frequencies were determined either including or excluding G-to-A hypermutants, with the results superimposed. The relative percentage of the total data that can be attributed (or not attributed) to G-to-A hypermutation is indicated within the *bars*. **b** Analysis of HIV-1 and HIV-2 mutation spectra in the absence of G-to-A hypermutation. HIV-1 and HIV-2 mutation spectra were examined after excluding all G-to-A hypermutants. Mutation spectra were determined by dividing the frequency of each type of mutation by the total mutation frequency, with the results expressed as a percentage. Data in *both panels* represent the mean of three experimental replicates.

The HIV-1 and HIV-2 mutational data was analyzed with and without G-to-A hypermutants in order to: (1) estimate the extent to which G-to-A hypermutation contributed to the total mutational data, and (2) determine whether G-to-A hypermutation was primarily responsible for observed differences between HIV-1 and HIV-2 mutation frequencies and spectra. While removal of G-to-A hypermutants had little effect on HIV-2 mutation frequencies (reducing the total mutation frequency by only 5%), their removal reduced the overall HIV-1 mutation frequency by 53% and the G-to-A transition mutation frequency by 81% (Figure 4a). Thus, G-to-A hypermutation was responsible for approximately half of all mutations observed in HIV-1, despite being a relatively rare event (~1 of 127 read pairs were G-to-A hypermutants). In the absence of G-to-A hypermutants, HIV-1 and HIV-2 total mutation frequencies were found to be 3.2 or 2.9 × 10^−4^ m/bp, respectively, a 1.1-fold difference that was not statistically significant (*p* = 0.14; Figure 4a). However, as noted earlier, HIV-2 displayed an ~1.1-fold lower frequency of A-to-G transitions (*p* = 0.049) and an ~1.5-fold higher frequency of C-to-T transitions (*p* = 0.0019) than HIV-1, and these findings were not altered by the removal of G-to-A hypermutants. Further, HIV-1 still demonstrated an ~1.8-fold higher frequency of G-to-A transitions (*p* < 0.001) than HIV-2 after removal of G-to-A hypermutants. As expected, the mutation spectra for HIV-1 and HIV-2 were also much more comparable in the absence of the G-to-A hypermutation data (compare Figures 4b, 2c), although HIV-1 displayed a somewhat higher percentage of G-to-A (27 vs. 17%) and lower percentage of C-to-T (14 vs. 23%) transitions than HIV-2. Taken together, these observations suggest that most of the observed differences in viral mutation patterns between HIV-1 and 2 were due to the highly reduced levels of G-to-A hypermutants for HIV-2. These findings imply that the fidelities of other mutational sources, such as RT, are relatively similar for HIV-1 and HIV-2.

## Discussion

Detection of rare mutations is often difficult with next-generation sequencing technologies due to high error rates, which for Illumina platforms typically range from ~10^−2^ m/bp for unfiltered data and ~10^−3^ to 10^−4^ m/bp for stringently filtered data [33–35, 46–49]. We adopted numerous measures to minimize background errors due to PCR and sequencing, as well as to minimize PCR-mediated recombination (see “Methods”). We also performed control amplifications from plasmids in which we matched conditions as closely as possible with the biological samples. After stringent filtering, we obtained error frequencies of 2.8 (HIV-1) or 2.6 (HIV-2) × 10^−4^ m/bp for the plasmid controls (Figure 2a). The frequencies and distribution of background errors were nearly identical between the HIV-1 and 2 plasmid controls (Figure 2a–c), as expected considering that the amplicons were either identical (HSA, EGFP-1, EGFP-2) or ~60% homologous (Gag, Vif) in sequence. Consistent with previous reports on Illumina background errors [33–36, 49], the spectra of background errors were more heavily biased toward transversions than biological samples (Figure 2c), specifically C-to-A and G-to-T transversions (Additional file 4: Figure S1). This trend was observed despite prior masking of plasmid error hotspots, which were also mostly (~83%) C-to-A and G-to-T transversions (see “Methods” and Additional file 10: Table S5). Two mechanisms for these transversion types have been suggested in the literature [33–36, 50]: (1) oxidative conversion of guanine to 8-oxoguanine during sample processing, which would lead to fixation of C-to-A and G-to-T mutations during PCR, and (2) imaging errors that result from crosstalk between the C and A channels or between the G and T channels, as the fluorophores attached to these bases are excited by the same lasers and then distinguished using different filters. Due to these issues, we unfortunately were not able to make a detailed comparison of transversion frequencies between HIV-1 and 2. However, transition frequencies [5.8 (HIV-1) and 2.0 (HIV-2) × 10^−4^ m/bp] were significantly higher than the plasmid controls for both viruses (HIV-1: *p* < 0.001; HIV-2: *p* = 0.038), and many previous reports have established transitions as the dominant type of mutations that occur during HIV-1 replication [1–4, 17, 19, 40]. Thus, further analyses focused primarily on these mutational types.

Relative to previous estimates of the HIV-1 mutation rate (which range from 1.4 to 8.5 × 10^−5^ mutations/bp/cycle) [1–5, 17, 41, 51], we obtained a somewhat higher mutation frequency for HIV-1 of 6.9 × 10^−4^ m/bp (or 5.8 × 10^−4^ m/bp if considering only transitions). However, these discrepancies may be due to several key differences between the assays used and the ways in which the data were analyzed. First, the level of background error in previous studies was likely much lower. Most previous estimates of the HIV-1 mutation rate were determined using the LacZα assay [1, 2, 4, 17, 41], in which a proviral *lacZα* sequence is purified (using the Lac repressor or Hirt extraction), directly transformed into *E. coli* (without using PCR), and subjected to blue/white color screening, followed by Sanger sequencing of mutants. While this assay results in little background, it is low-throughput and can only detect and measure mutations within the *lacZα* marker gene. Second, previously used marker gene assays (such as the LacZα assay) necessarily underestimate true mutation rates because they only detect mutations leading to a phenotypic change (silent mutations are not detected unless co-occurring with other mutations). For the LacZα assay, measured mutation rates are thought to underestimate actual mutation rates by ~2 to 3-fold [4]. In contrast, the assay we used based on direct PCR and Illumina sequencing of multiple amplicons should detect all mutations, provided that they do not prevent amplification. Third, most previous studies used a second marker gene (typically an antibiotic resistance gene) to select for infected cells or to select for transformed *E. coli* [1–4, 17, 41, 51]. This approach will not detect heavily mutated sequences (such as hypermutants) in which both marker genes are mutated and functionally disrupted. Fourth, actual mutation frequencies may vary between the genes analyzed here (*gag*, *vif*, *hsa*, and *egfp*) and mutational targets from previous studies. Fifth, many previous studies scored mutants harboring multiple mutations as single mutants for the purposes of calculating mutation rates [1, 2, 4, 17, 41, 51]. Also, in some cases background error frequencies were subtracted from the mutation frequencies of the biological samples in order to calculate mutation rates [5].

Unexpectedly, we identified G-to-A hypermutants for both HIV-1 and 2, though the frequency was much higher (~28-fold) for HIV-1 than HIV-2 (Figure 3a; *p* < 0.001). The presence of G-to-A hypermutants could not be attributed to background errors, as their frequencies were much higher in the biological samples than in the plasmid controls. Also, the patterns of G-to-A hypermutation (in terms of dinucleotide context preferences and hypermutation hotspots) were markedly distinct between the biological samples and plasmid controls (Figure 3d and data not shown). Unlike single G-to-A mutants from the biological samples, G-to-A hypermutants exhibited a strong bias toward GA dinucleotide contexts (Figure 3d; Additional file 8: Figure S3). In sum, these findings support the idea that the G-to-A hypermutants were caused by low-level activity of one or more APOBEC3 proteins. However, APOBEC3G cannot be primarily responsible, due to its strong preference for GG dinucleotides [42–44, 52]. Further experiments in which specific APOBEC3 proteins are down-regulated (through knockout or knockdown) are required to prove that the G-to-A hypermutants we observed are APOBEC3-mediated, as well as to identify the specific APOBEC3 protein(s) responsible.

The observation of G-to-A hypermutation was somewhat surprising because: (1) the HIV-1 and HIV-2 vectors used in these studies encoded for a functional Vif protein, (2) the expression levels of most APOBEC3 proteins are relatively low in 293T (i.e., the cells that produced the HIV-1 and HIV-2 vector viruses) [19], and (3) Vif-proficient and Vif-deficient viruses exhibit similar infectivities when produced in 293T cells [13, 15, 32, 53]. Upon observing G-to-A hypermutation, we confirmed via Sanger sequencing that the *vif* genes in the HIV-1 and HIV-2 vectors were intact (data not shown). Notably, previous studies have demonstrated that Vif does not always fully neutralize the activity of APOBEC3 proteins [13, 42, 54–56]. In addition, Vif from different subtypes or containing naturally occurring polymorphisms have been shown to vary widely in their neutralization capacities [55–57]. Further, multiple APOBEC3 proteins (including B, C, D, and F) have been detected at the mRNA level in 293T cells and could be involved in the observed G-to-A hypermutation [19, 58]. Previous studies by our group have identified rare G-to-A hypermutants occurring at GA dinucleotides by Sanger sequencing of clones (in which case the virus stocks were also generated in 293T cells) [19, 59]. Taken together, the G-to-A hypermutation observed here was likely due to the failure of HIV-1 or HIV-2 Vif to fully neutralize low levels of APOBEC3 proteins present in 293T producer cells. Consistent with this hypothesis, G-to-A hypermutation was observed very infrequently even for HIV-1 (~1 of 127 read pairs were G-to-A hypermutants), despite contributing to about half of all mutations (Figure 4a). Although HIV-2 was less susceptible than HIV-1 to G-to-A hypermutation in this experimental system, further investigation will be required to determine whether these trends hold true under conditions of higher APOBEC3 expression. Intriguingly, HIV-2 ∆*vif* has been reported to be less sensitive than HIV-1 ∆*vif* to APOBEC3G [32], but susceptibilities to other APOBEC3 proteins (in the presence or absence of Vif) have not yet been compared. Further, HIV-1 and HIV-2 Vif were recently shown to interact with APOBEC3F and APOBEC3G through completely separate sequence determinants, and differences in HIV-1 and HIV-2 Vif-induced degradation of specific APOBEC3 proteins were also noted [60].

Most of the G-to-A hypermutants occurred within the EGFP-1 and Vif amplicons (Figure 3b). This may be due in part to higher frequencies of GA dinucleotides in these particular amplicons. For HIV-1, the frequencies of GA dinucleotides (relative to GG, GT, and GC) were 13% (Gag), 32% (Vif), 9% (HSA), 32% (EGFP-1), and 30% (EGFP-2). Thus, the GA dinucleotide frequency varied maximally by 3.6-fold, whereas G-to-A hypermutation frequencies varied up to 26.3-fold (EGFP-1 vs Gag). However, APOBEC3-mediated hypermutation can also be influenced by broader sequence contexts and secondary structures [14, 15, 42, 44, 61–63]. Further, APOBEC3 activity follows a twin gradient along the HIV-1 genome corresponding to the amount of time the minus strand viral DNA remains single-stranded, such that the positioning of the amplicon could affect hypermutant frequencies [64, 65]. Thus, one or more of these other features may have favored hypermutation in the EGFP-1 and Vif amplicons. Full genome sequencing will be required to address the distribution of G-to-A hypermutation in more detail. G-to-A hypermutation hotspots were also identified (Additional file 9: Table S4), which all occurred in the EGFP-1 and Vif amplicons. G-to-A hotspots did not result in any nonsense mutations, consistent with the notion that GA dinucleotide-biased hypermutation generates fewer stop codons than GG-biased hypermutation. However, the G-to-A hotspots did introduce a number of missense mutations, particularly D-to-N, E-to-K, and R-to-K. Surprisingly, all of the mutations resulting from the four hotspots in HIV-1 Vif were recently identified in another study in which humanized mice were infected with an HIV-1 variant that cannot neutralize APOBEC3D or F [15]. One of these Vif mutants (E134K) lost the ability to neutralize APOBEC3G, raising the possibility that hypermutation itself can influence susceptibility to hypermutation in further rounds of replication.

We initially hypothesized that HIV-2 would exhibit a lower mutation frequency than HIV-1 due to its attenuated pathogenicity, reduced evolutionary rate [27, 28], a conserved fidelity-improving V75I polymorphism in RT [30, 31], and reduced susceptibility to APOBEC3G [32]. More specifically, HIV-2 Env has been found to diversify more slowly than HIV-1 Env when compared to HIV-1-infected individuals with high viral loads [28]. However, HIV-1 and HIV-2 diversification rates were similar when compared to HIV-1-infected individuals with low viral loads. In the same report, HIV-2 Env was found to evolve more slowly than HIV-1 Env and to be subject to strong purifying selection. Indeed, HIV-2 infection appears to elicit broad and potent neutralizing antibody responses against Env more frequently than for HIV-1 [66]. In another report, HIV-2 Env was found to exhibit a lower rate of synonymous substitutions than HIV-1, implying reduced viral mutation and/or replication rates [27], though another group has reported opposing findings [29]. Unfortunately, it is difficult to compare the results of our analyses to these published reports due to these contradicting results, and larger studies of HIV-2 intra-patient diversification and evolution are clearly warranted. Nonetheless, we found that HIV-1 and HIV-2 exhibited similar total mutation frequencies in the absence of G-to-A hypermutants, suggesting that differences in replication fidelity do not have a major impact on differences in evolutionary or synonymous substitution rates between HIV-1 and HIV-2.

## Conclusions

In sum, we have found that HIV-2 exhibited significantly lower total and transition mutation frequencies than HIV-1, as well as a mutation spectrum less biased toward G-to-A transitions. However, these differences were mostly due to a significantly higher G-to-A hypermutation frequency for HIV-1 than HIV-2. These findings raise the intriguing possibility that HIV-2 might be less sensitive than HIV-1 to APOBEC3-mediated hypermutation, consistent with a previous report [32], but additional experiments in other cell types will be required to fully address this question. After removal of all G-to-A hypermutants, HIV-1 and HIV-2 total mutation frequencies were not significantly different, although small but significant differences were still observed in the frequencies of G-to-A and C-to-T transitions. Overall, these results suggest that the fidelities of other mutagenic processes (such as reverse transcription) are relatively similar between the two viruses. Nevertheless, we cannot rule out the possibility that HIV-1 and HIV-2 exhibit more minor differences in mutation frequencies or spectra that we were not able to detect or that differences would be observed in other cell types. Overall, these data imply that differences in replication fidelity are likely not a major contributing factor to the unique clinical features of HIV-2 infection.

## Methods

### Plasmids, cell lines, and reagents

The HIV-1 vector, pNL4-3 HIG, has been previously described [67]. This vector contains a cassette encoding heat stable antigen (HSA), an internal ribosomal entry site (IRES), and enhanced green fluorescent protein (EGFP). The HIV-2 vector, pROD HIG, was created from pHIV-2 H0G [68], a kind gift from Dr. Wei-Shau Hu (HIV Drug Resistance Program, Frederick National Laboratory for Cancer Research, Frederick, MD, USA). The pHIV-2 H0G vector does not express Vpr or EGFP due to multiple point mutations. In order to construct pROD HIG, the *vpr* and *egfp* genes were restored by site-directed mutagenesis using the QuikChange II XL kit (Agilent Technologies, Inc.; Santa Clara, CA, USA), and the resulting vector was verified by DNA sequencing. Both HIV-1 and HIV-2 vectors contain intact open reading frames for all genes except for the *env* and *nef* genes. Vector viral stocks from pNL4-3 HIG were produced by co-transfecting with pNL4-3 Env, a kind gift from Dr. Eric Freed (HIV Drug Resistance Program, Frederick National Laboratory for Cancer Research, Frederick, MD, USA). Vector viral stocks of pROD HIG were produced by co-transfecting pROD10-Env [69], a kind gift from Dr. Paula Cannon (University of Southern California, Los Angeles, CA, USA). The human embryonic kidney (HEK 293T) cells were purchased from American Type Culture Collection (Manassas, VA, USA) and maintained in Dulbecco’s Modified Eagle’s Medium (DMEM) from Cellgro (Manassas, VA, USA) with 10% HyClone FetalClone III (FC3) from Thermo Scientific (Waltham, MA, USA) and 1% penicillin/streptomycin from Life Technologies (Grand Island, NY, USA). U373-MAGI-CXCR4_CEM_ cells were obtained from Dr. Michael Emerman through the NIH AIDS Reagent Program, Division of AIDS, NIAID, NIH [70]. U373-MAGI cells were maintained similarly to 293T cells but with addition of 1.0 μg/mL puromycin, 0.1 mg/mL hygromycin B, and 0.2 mg/mL G418 to the medium. For transfections, poly-l-lysine was from Newcomer Supply (Middleton, WI, USA) and polyethylenimine (PEI) was from Polysciences, Inc. (Warrington, PA, USA).

### Virus production and titering

Virus was produced by co-transfecting pNL4-3 HIG or pROD HIG with pNL4-3 Env or pROD10 Env, respectively, into 293T cells via the PEI method [71]. PEI stocks were prepared at 1 mg/mL by dissolving PEI in water, adjusting the pH to 7.0, and filtering through a 0.2 µm filter. 24 h before transfection, 4 million 293T cells/plate were seeded onto 10 cm plates pre-coated for 5 min with poly-l-lysine. For each plate, 10 µg of pNL4-3 HIG or pROD HIG + 5 µg Env expression plasmid + 45 µL 1 mg/mL PEI were combined with serum-free DMEM to a final volume of 1 mL. After 20 min of incubation, the medium on the 293T cells was replaced and the DNA-PEI mixture was added. The medium was replaced 16 h post-transfection, and viral stocks were collected 48 h post-transfection by filtering the supernatants through a 0.2 µm filter. For each viral stock, five plates were transfected, and the resulting supernatants were pooled and concentrated (~tenfold) using 100,000 MWCO filtration columns (Vivaproducts; Littleton, MA, USA). Viral stocks were then treated with 10 U/mL of DNase I (New England Biolabs; Ipswich, MA, USA) for 2 h at 37°C to degrade residual plasmid DNA from transfections. Viral stocks were then divided into 1.0 mL aliquots and frozen at −80°C.

Prior to large-scale infections, viral stocks were first titered in U373-MAGI cells based on EGFP expression. The day before infection, 31,250 cells/well were plated in 24-well plates. After 24 h, the media was replaced and varying volumes of virus ranging from 15.625 to 500 µL (twofold dilution series) were added. To improve infectivity, the cells were infected by spinoculation (1,200 × g for 2 h at 24°C). The media was replaced again 24 h post-infection and cells were collected at 72 h post-infection for analysis of EGFP expression by flow cytometry. The cells were treated with trypsin, transferred to 96-well plates, pelleted at 500 × g for 5 min, and resuspended in 200 µL Dulbecco’s phosphate-buffered saline (DPBS) + 2% FC3/well. EGFP expression from at least 10,000 gated cells was analyzed using a BD LSR II flow cytometer (BD Biosciences; San Jose, CA, USA). EGFP was excited with a blue 488-nm laser and emission detected using 505LP and 525/50 filters. Virus titers (expressed as infectious units/mL) were calculated based upon EGFP expression at low infectivities (<40%) as previously described [72].

### Infections for Illumina sequencing

In order to prepare samples for Illumina sequencing, 1 × 10^6^ U373-MAGI cells were infected at a multiplicity of infection (MOI) of 1.0. These infections were performed in 24-well plates (31,250 cells/well) in order to avoid any potential effect of the plate format on infectious titer. Uninfected cells and cells infected with heat-inactivated viruses (i.e. virus stocks that were incubated at 65°C for 1 h) were included as negative controls. The cells were infected by spinoculation, and the medium was replaced 24 h post-infection. Cells were collected for genomic DNA extraction at 72 h post-infection by treating with trypsin, pelleting, and washing three times with DPBS to further reduce plasmid carryover. Extra wells of infected cells were analyzed by flow cytometry to verify infectivity. In this assay, all proviruses result from a single cycle of infection, as neither producer cells nor target cells can be re-infected, due to a lack of the appropriate receptor and co-receptor or to a lack of envelope expression, respectively (Figure 1).

### Genomic DNA extraction and quantification of plasmid carryover

Genomic DNA was extracted from all collected cells using the High Pure PCR Template Preparation Kit (Roche; Basel, Switzerland) following the manufacturer’s instructions and eluted in 150 μL buffer. Genomic DNA was treated with *Dpn*I for 1 h at 37°C to further reduce plasmid carryover from the transfections, after which *Dpn*I was heat-inactivated at 80°C for 20 min. In order to quantify any residual plasmid carryover, two approaches based on quantitative PCR (qPCR) were adopted: (1) the ampicillin resistance gene copy number was determined and divided by the proviral copy number (as measured using the HSA Illumina amplicon), or (2) the proviral copy number from heat-inactivated virus infections was determined and divided by the proviral copy number from the corresponding un-treated infections. For both approaches, qPCR was performed using 4 μL water, 6.25 μL 2× Power SYBR Green Master Mix (Life Technologies), 0.625 μL each primer (500 nM final concentration), and 1 μL template. The cycling conditions used were an initial denaturation of 95°C 10 m, 40 cycles of 95°C 15 s/55°C 15 s/72°C 30 s, and a final extension of 72°C 7 min. The sequences of the primers to the ampicillin resistance gene were 5′-ACTTTATCCGCCTCCATCCAGTC-3′ and 5′-GAGCGTGACACCACGATGC-3′. Absolute standard curve series (from 10^1^ to 10^6^ copies/μL) were constructed by quantifying the pNL4-3 HIG and pROD HIG plasmids with the Qubit dsDNA HS Assay Kit (Life Technologies). We found that the level of plasmid carryover for HIV-1 was 0.2% when measured by either method, while the level of carryover was 2.8 or 1.4% for HIV-2, depending on the approach used (Additional file 1: Table S1). These results are comparable to those obtained in a similar study of HIV-1 mutagenesis [5] and are too low to significantly affect measured mutation frequencies.

### Amplifications for Illumina sequencing

PCR was performed on five small (~150–170 bp) amplicons (Gag, Vif, HSA, EGFP-1, EGFP-2) for each sample, with HIV-1 and 2 amplicons positioned in homologous locations. All forward primers contained 5-base barcodes (differing by at least two bases) to allow demultiplexing of pooled PCR products. All primer and barcode sequences are listed in Additional file 11: Table S6. Barcodes were randomly generated using a program written by Luca Comai and Tyson Howell (http://comailab.genomecenter.ucdavis.edu/index.php/Barcode_generator) [73]. PCR was performed using the Phusion Hot Start II High-Fidelity DNA Polymerase (Fisher Scientific; Pittsburgh, PA, USA). PCR reactions were performed with 8 µL of genomic DNA (~50,000 target copies), 500 nM of each primer, and a final volume of 50 µL/reaction. The cycling conditions used were an initial denaturation of 98°C 30 s, 30 cycles of 98°C 10 s/56°C 30 s/72°C 15 s, and a final extension of 72°C 10 min. All PCR reactions were performed in triplicate to reduce the risk of clonal amplification, and the products were pooled after amplification. Negative control reactions were performed lacking template or with genomic DNA from uninfected cells.

In order to assess the degree of background error due to PCR and Illumina sequencing, control amplifications were performed from pNL4-3 HIG or pROD HIG plasmids using the same cycling conditions. For these reactions, 50,000 copies of plasmid per 50 µL reaction was used, a target level found via qPCR to be similar to that of the other samples. Genomic DNA (8 µL/reaction) from uninfected cells was added to the plasmid PCR reactions to account for any potential impact of genomic DNA on amplification. Further, the degree of intra-sample PCR-mediated recombination was investigated in the plasmid controls, as recombination could affect the association between mutations and thus alter hypermutant frequencies. To determine recombination frequencies, EGFP-1 was amplified from a 1:1 mixture (i.e. 25,000 copies each) of wild-type and mutant EGFP-1 sequences. The mutant EGFP-1 sequence contained two genetic markers positioned ~50 bp apart, and each marker consisted of two mutations to facilitate distinction from PCR and sequencing-induced errors. Intra-sample recombinants were detected by identifying read pairs from the plasmid controls with a single marker set, rather than the expected zero or two marker sets present in the wild-type and mutant EGFP-1 sequences, respectively. Importantly, all mutations utilized as recombination markers were masked before mutational analysis.

### Library preparation and Illumina sequencing

Amplicons were gel-purified using the Promega SV Gel Extraction Kit (Promega Corp.; Madison, WI, USA). After gel purification, all amplicons were quantified using the Qubit dsDNA HS Assay Kit (Life Technologies) and a Qubit 2.0 fluorometer. For each sample, all amplicons were pooled together in an equimolar fashion to normalize coverage between amplicons. Next, 100 ng of each sample (12 samples in total) were submitted to the University of Minnesota Genomics Center for library preparation. The libraries were constructed with the TruSeq Nano DNA Sample Preparation Kit following the manufacturer’s instructions but using AMPure XP beads (Beckman Coulter, Inc.; Indianapolis, IN, USA) at a bead to sample ratio of 1.8. The 15 libraries were again quantified using the Qubit dsDNA BR Assay Kit, size-confirmed with Agilent DNA 1000 chips (Agilent Technologies; Santa Clara, CA, USA), and pooled in an equimolar fashion to normalize coverage between libraries. Samples were pooled after library preparation, rather than before, because it had been previously determined that significant inter-sample recombination can occur during the 10 cycles of PCR typically utilized in library construction (data not shown). In order to improve sequence diversity and quality, a PhiX library was added in at ~25% of total mass. Next, 2 μL of the 10 nM library pool was denatured and diluted to a final concentration of 4 pM for DNA sequencing. Sequencing of proviral DNA was conducted by using the Illumina MiSeq with 2 × 150 paired-end sequencing. All Illumina sequencing data supporting the results of this manuscript have been deposited into the NCBI Sequence Read Archive (SRA) under accession code BioProject PRJNA287455.

### Processing of proviral DNA sequencing data

First, paired-end reads from the Illumina MiSeq reaction were demultiplexed based on perfect barcode matches, and barcode sequences were trimmed off during the process. Second, poor quality reads were filtered out using quality criteria found to reduce Illumina background error rates [35]. Specifically, read pairs were discarded in which either read contained a B-tail (i.e. one or more low quality bases at the end of the read), contained at least one uncalled base, had less than two-thirds of bases with Q-score ≥30 in the first half of the read, or had an average Q-score <30 in the first 30% of the read. Third, reads from each of the 12 samples were mapped to the appropriate reference sequence (pNL4-3 HIG or pROD HIG) with GSNAP [74] using default parameters. Finally, a small number of read pairs (~35,000) that were aligned either partially or fully outside of the appropriate amplicon regions were excluded. For each sample, the numbers of initial read pairs, read pairs lost during mapping or filtering, and final read pairs are listed in Additional file 2: Table S2. We obtained ~4.7 million total read pairs after all processing steps, which removed primarily PhiX read pairs or HIV read pairs with imperfect barcodes or poor quality. About 319,000–461,000 read pairs were obtained per sample (average of 395,000 read pairs/sample), or 46,000–111,000 read pairs per amplicon per sample (average of 79,000 read pairs/amplicon/sample).

In order to identify mutations present in read pairs passing the above processing steps, a custom algorithm was developed to compile mutation frequency data for each sample, built using the Genome Analysis Toolkit (GATK) walker framework [75]. This algorithm determines both the frequency of total mutations as well as of specific mutational types (substitutions, transitions, transversions, every type of transition or transversion, insertions, deletions, etc.). In order to minimize background error rates, mutations were required to be identified on both sequences in a read pair, which was possible because forward and reverse reads were mostly overlapping due to small amplicon sizes (~150–170 bp). Furthermore, substitutions and insertions were only counted if they had a Q-score ≥30 for the relevant base(s) on both reads. Wild-type bases had to meet the same criteria as mutations (i.e. identified as wild-type and Q-score ≥30 on both sequences of a read pair). Non-overlapping segments of read pairs as well as single reads were excluded from mutational analyses. All primer sequences were also excluded from mutational analysis, as errors within primers would not represent biologically meaningful mutations. We also examined the distribution of all background errors (from PCR and sequencing) in the plasmid controls and identified numerous plasmid error hotspots (defined as upper outliers within the distribution of frequencies of individual mutations based on the 1.5 × IQR rule). Most plasmid error hotspots (~83%) were G-to-T or C-to-A transversions. Within identical amplicons (HSA/EGFP-1/EGFP-2), many common plasmid error hotspots were identified in the HIV-1 and HIV-2 plasmid controls (~88% overlap), whereas the degree of overlap was much lower for the ~60% homologous viral amplicons (~49% overlap in Gag and ~31% overlap in Vif). Plasmid error hotspots that were shared between the HIV-1 and HIV-2 plasmid controls (Additional file 10: Table S5) were masked before further downstream analysis, as the presence of these mutations in the biological samples would most likely represent background errors. Rather than masking all mutational types at error hotspots, only the problematic type(s) (e.g. G-to-T) were masked at the indicated positions. Insertions and deletions were scored as single events regardless of the number of bases inserted or deleted. Mutation frequencies (defined as mutations/bp) were calculated by dividing the number of mutations passing filters by all reference bases (mutations + wild-type bases) passing filters.

In addition to examining mutation frequencies and spectra, hypermutants were identified and characterized within the Illumina sequencing data. Using a custom GATK walker, hypermutant counts for each type of transition were collected (G-to-A, A-to-G, T-to-C, and C-to-T). Hypermutants were defined as individual read pairs containing two or more of the same type of transition. All transitions had to be identified on both sequences with Q-scores ≥30, and a single read pair could theoretically count as two different types of hypermutants if it contained multiple instances of two different transition types. Hypermutant frequencies were calculated by dividing the number of hypermutant read pairs by all (hypermutant + non-hypermutant) read pairs that passed the processing steps. In order to examine G-to-A hypermutation hotspots, ranked lists of G-to-A mutation frequencies were generated at individual bases within G-to-A hypermutants. Hypermutation hotspots were then defined as statistical upper outliers within the distribution of frequencies using the 1.5 × IQR rule.

### Biostatistical analysis of Illumina DNA sequencing data

To test for factors that may influence mutation frequencies, generalized linear mixed effects models were applied to the data that came from our Illumina data processing pipeline. The raw counts for each type of mutation (e.g. transitions) were modeled as Poisson random variables with an offset given by the total number of reference bases. The type of sample (i.e. HIV-1, HIV-2, and plasmid controls for HIV-1 and HIV-2), the type of amplicon, and their interactions were treated as fixed effects while the replicate was treated as a random effect. The logarithmic link was used, as is standard for Poisson outcomes, and penalized quasilikelihood was used to estimate the model parameters [76]. These computations were conducted using R v 3.1.0 and the MASS package. All figures and tables were created in GraphPad Prism v 6.0 (GraphPad Software, Inc.; La Jolla, CA, USA) or Microsoft Office for Mac 2011 v 14.3.8 (Microsoft Corp.; Redmond, WA, USA).

## Additional files

Additional file 1:
**Table S1.** Quantification of plasmid carryover from virus production. The levels of HIV-1 or HIV-2 plasmids from transfections remaining in genomic DNA samples were determined using quantitative PCR-based approaches. Additional file 2:
**Table S2.** Output of 2 × 150 Illumina sequencing of HIV-1 and HIV-2 after data processing. For each sample, the numbers of read pairs before and after quality filtering are indicated, both grouped across all amplicons and separated by amplicon. Additional file 3:
**Dataset S1.** HIV-1 and HIV-2 mutation frequencies determined by Illumina sequencing. For each sample, this dataset lists the numbers of reference bases, mutations, mutation frequencies, and relative percentages of many different mutational types. The data is shown both grouped across all amplicons and separated by amplicon (as different tabs within the spreadsheet). Additional file 4:
**Figure S1.** Bias of transversion spectra toward C-to-A and G-to-T mutations. The transversion spectra for HIV-1 and HIV-2 biological samples and plasmid controls are illustrated, revealing a clear bias toward C-to-A and G-to-T transversion types, particularly for the plasmid controls. Additional file 5:
**Table S3.** Determination of intra-sample recombination frequencies from PCR. The frequency of intra-sample recombination resulting from PCR was determined using a set of genetic markers that were incorporated into the HIV-1 and HIV-2 plasmid controls. Additional file 6:
**Dataset S2.** HIV-1 and HIV-2 hypermutant frequencies determined by Illumina sequencing. For each sample, this dataset lists the numbers of total read pairs, hypermutant read pairs, and hypermutation frequencies for all four possible types of transition hypermutants (G-to-A, A-to-G, C-to-T, T-to-C). Hypermutants were defined as read pairs (~ 120 bp in length) containing two or more of the same type of mutation. The data is shown both grouped across all amplicons and separated by amplicon (as different tabs within the spreadsheet). Additional file 7:
**Figure S2.** Transition frequencies are similar across amplicons in the absence of G-to-A hypermutants. The frequencies of transitions were determined in all five amplicons after exclusion of all G-to-A hypermutants (individual read pairs containing two or more G-to-A mutations). Additional file 8:
**Figure S3.** Dinucleotide contexts of G-to-A mutations not occurring within G-to-A hypermutants. The dinucleotide contexts of all G-to-A mutations from HIV-1 and HIV-2 single mutants (i.e. non-hypermutants) were determined, demonstrating the lack of a bias toward GA and/or GG dinucleotides. Additional file 9:
**Table S4.** Identification of G-to-A hypermutation hotspots in HIV-1 and HIV-2. G-to-A hypermutation hotspots were identified as statistical upper outliers according to the 1.5 × IQR rule. For each hotspot, the relative ranking, position, dinucleotide context, mutation frequency, and coding effects are indicated. Additional file 10:
**Table S5.** Identification and masking of plasmid error hotspots. Plasmid error hotspots (i.e. common sites for background errors due to PCR or sequencing) were identified by examining the distribution of mutation frequencies at every individual position for the HIV-1 and HIV-2 plasmid controls. Plasmid error hotspots were defined as upper outliers within the distribution using the 1.5 × IQR rule. Hotspots identified in both HIV-1 and HIV-2 plasmids were masked from all samples prior to mutational analyses, as they would most likely represent background errors in the biological samples. Additional file 11:
**Table S6.** HIV-1 and HIV-2 primer and barcode sequences for Illumina sequencing.
