# Supplementary material for: HIV-1 and HIV-2 exhibit similar mutation frequencies and spectra in the absence of G-to-A hypermutation
Source: Retrovirology. 2015 Jul 10;12:60. doi: 10.1186/s12977-015-0180-6 (PMC4496919; doi:10.1186/s12977-015-0180-6)
Supplement: Additional file 4: — Figure S1. Bias of transversion spectra toward C-to-A and G-to-T mutations. The transversion spectra for HIV-1 and HIV-2 biological samples and plasmid controls are illustrated, revealing a clear bias toward C-to-A and G-to-T transversion types, particularly for the plasmid controls. [file 12977_2015_180_MOESM4_ESM.pptx]

## Slide 1
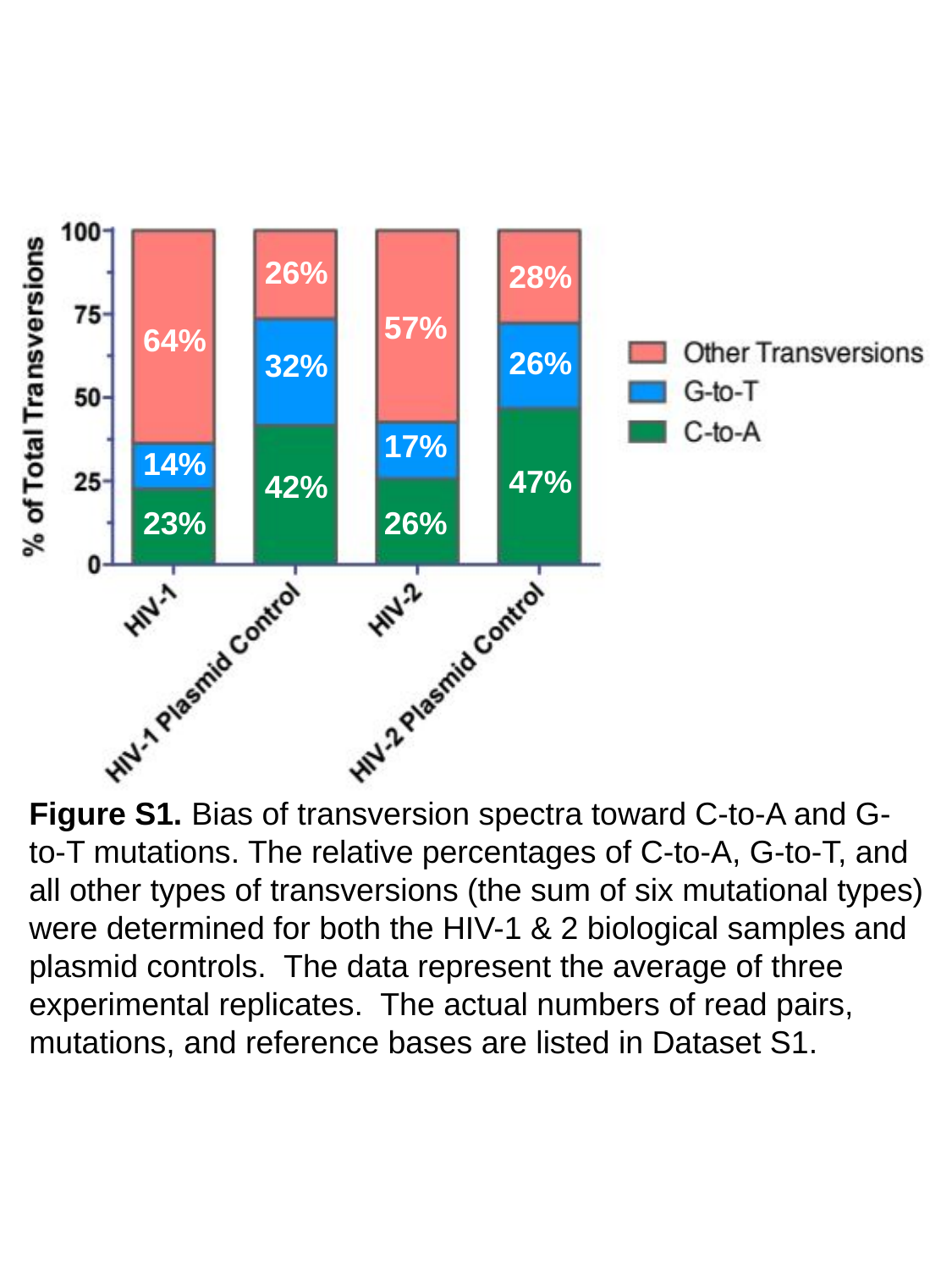

26%
28%
57%
64%
26%
32%
17%
14%
47%
42%
23%
26%
Figure S1. Bias of transversion spectra toward C-to-A and G-to-T mutations. The relative percentages of C-to-A, G-to-T, and all other types of transversions (the sum of six mutational types) were determined for both the HIV-1 & 2 biological samples and plasmid controls. The data represent the average of three experimental replicates. The actual numbers of read pairs, mutations, and reference bases are listed in Dataset S1.
