# Supplementary material for: HIV-1 and HIV-2 exhibit similar mutation frequencies and spectra in the absence of G-to-A hypermutation
Source: Retrovirology. 2015 Jul 10;12:60. doi: 10.1186/s12977-015-0180-6 (PMC4496919; doi:10.1186/s12977-015-0180-6)
Supplement: Additional file 7: — Figure S2. Transition frequencies are similar across amplicons in the absence of G-to-A hypermutants. The frequencies of transitions were determined in all five amplicons after exclusion of all G-to-A hypermutants (individual read pairs containing two or more G-to-A mutations). [file 12977_2015_180_MOESM7_ESM.pptx]

## Slide 1
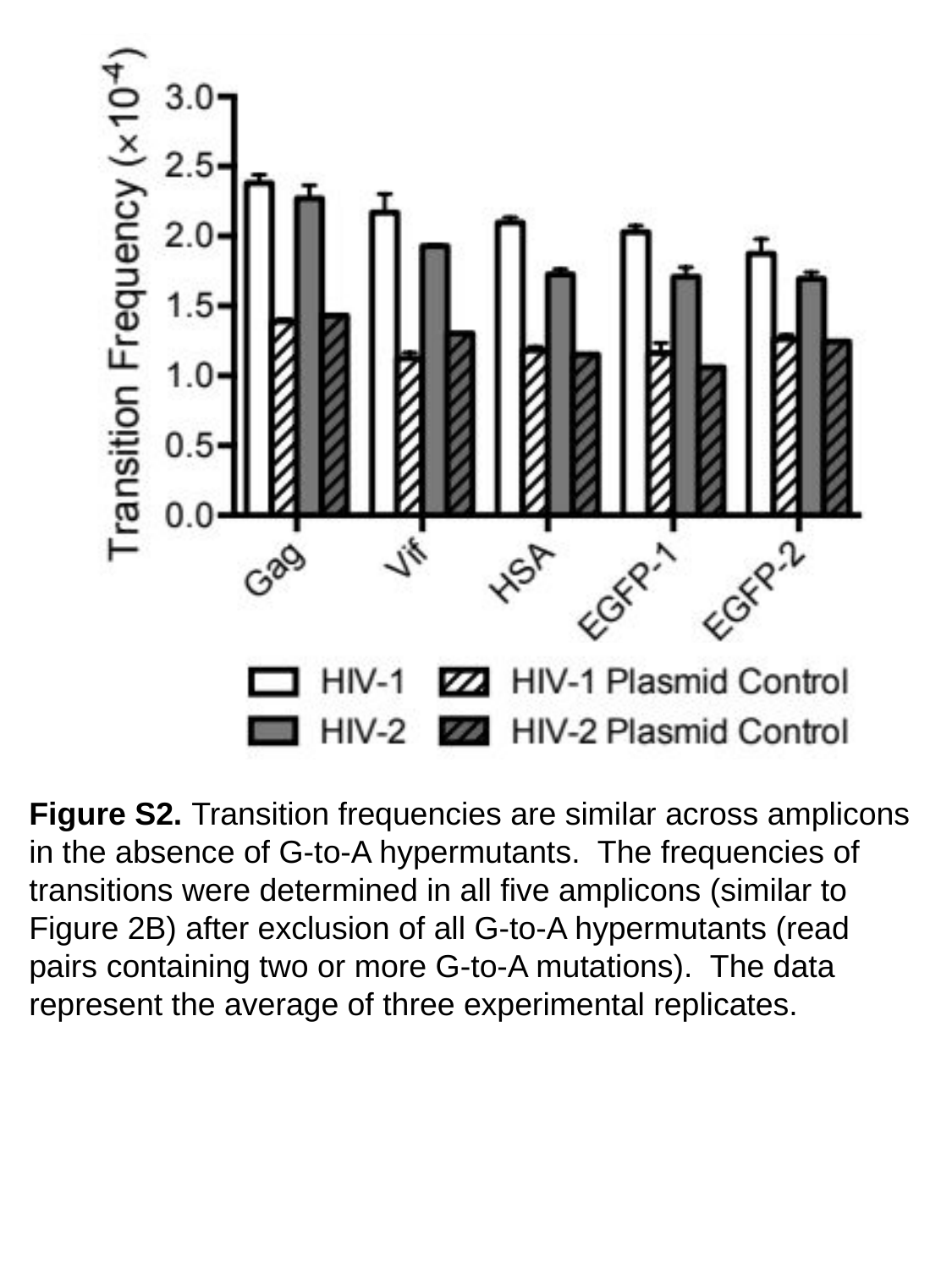

Figure S2. Transition frequencies are similar across amplicons in the absence of G-to-A hypermutants. The frequencies of transitions were determined in all five amplicons (similar to Figure 2B) after exclusion of all G-to-A hypermutants (read pairs containing two or more G-to-A mutations). The data represent the average of three experimental replicates.
