# Supplementary material for: HIV-1 and HIV-2 exhibit similar mutation frequencies and spectra in the absence of G-to-A hypermutation
Source: Retrovirology. 2015 Jul 10;12:60. doi: 10.1186/s12977-015-0180-6 (PMC4496919; doi:10.1186/s12977-015-0180-6)
Supplement: Additional file 8: — Figure S3. Dinucleotide contexts of G-to-A mutations not occurring within G-to-A hypermutants. The dinucleotide contexts of all G-to-A mutations from HIV-1 and HIV-2 single mutants (i.e. non-hypermutants) were determined, demonstrating the lack of a bias toward GA and/or GG dinucleotides. [file 12977_2015_180_MOESM8_ESM.pptx]

## Slide 1
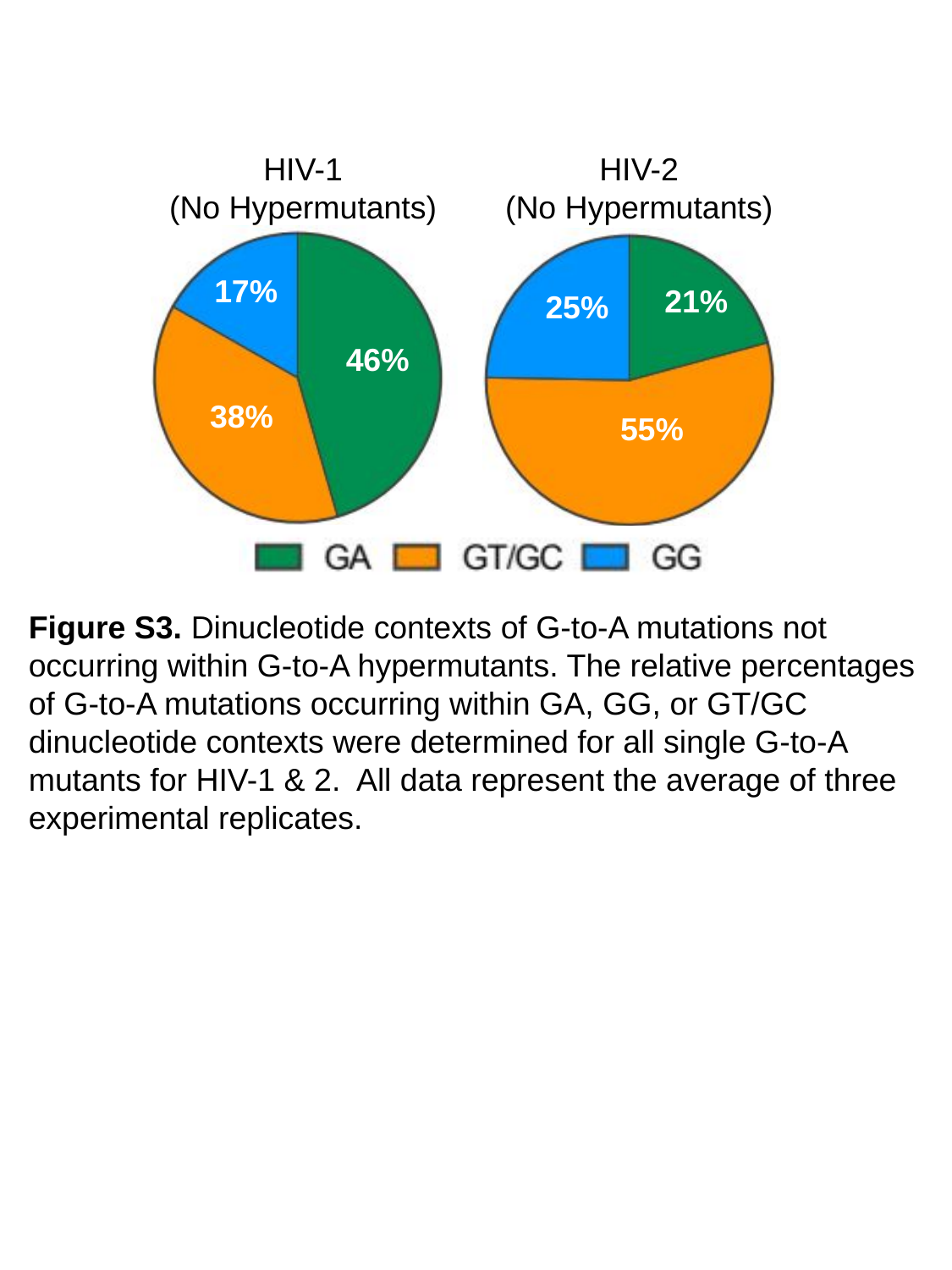

HIV-1
(No Hypermutants)
HIV-2
(No Hypermutants)
17%
21%
25%
46%
38%
55%
Figure S3. Dinucleotide contexts of G-to-A mutations not occurring within G-to-A hypermutants. The relative percentages of G-to-A mutations occurring within GA, GG, or GT/GC dinucleotide contexts were determined for all single G-to-A mutants for HIV-1 & 2. All data represent the average of three experimental replicates.
